# Some Practical Notes

**Published:** 1909-12-25

**Authors:** 


### SOME PRACTICAL NOTES.

During the cold weather it is often possible to economise petrol by removing the belt from the radiator or by shielding a portion of the radiator from the air by means of a covering of some sort. This prevents the circulating water from being maintained at as low a temperature as it otherwise would be, and reduces the losses through the cylinder walls, thus increasing motor efficiency.

The generators of acetylene lamps should never be filled with hot water (from the radiator, for instance), or, if this is necessary, one should wait until the water cools before it is fed to the carbide. The latter is not decomposed in the same way by warm water as by cold. It gives rise to "polymers" of acetylene, in the form of very light, light-black or yellow powders, which are drawn into the tubing and to the burner-tips, and very easily choke them.

A tantalising, but not infrequent cause of puzzling intermittence in the running of a petrol motor is a floating particle, such as a thin flake of solder which has been detached from the carburettor, or has entered the float chamber from the tank. Carried in the intermittent current of the liquid, it may suddenly become lodged so as to cover the duct to the nozzle, preventing all egress of petrol. When the motor stops the petrol in the float chamber ceases to be stirred, and the particle finds a new position. When the motor is started again it is not in the way, but presently it returns and again stops the flow of spirit. Should engine troubles be experienced the float chamber of the carburettor should therefore always be included in the parts to be inspected.

The springs of a car should be examined occasionally, for while they are often overlooked, this seemingly trifling matter has a direct bearing on the smooth and easy running of the vehicle. Owing to the fact that springs are exposed to the weather, rust is very likely to occur, and to this unsuspected corrosion is often due the occasional squeak which results. Although some motor-cars are provided with means for lubricating the friction surfaces, many are not so well provided for, and when rust makes its appearance along the joints there is a crying need for oil. The shackle joints should also have a little oil occasionally. The steering-gear should always be given proper care, and the levers, pins, and joints should be kept free of dirt, and well oiled. This is a very important matter, and as neglect may result in a bad accident, this important part of the mechanism should be given frequent and critical examination.

"Viator."

